# Psychosocial model of burnout among humanitarian aid workers in Bangladesh: role of workplace stressors and emotion coping

**DOI:** 10.1186/s13031-023-00512-1

**Published:** 2023-04-03

**Authors:** Cheryl Yunn Shee Foo, Alvin Kuowei Tay, Yexinyu Yang, Helen Verdeli

**Affiliations:** 1 Department of Counseling and Clinical Psychology, Teachers College, Columbia University of New York, 525 W 120 St, Box 102, New York, NY 10027 USA; 2 MGH Center of Excellence for Psychosocial and Systemic Research, Department of Psychiatry, Massachusetts General Hospital, Harvard Medical School, 1 Bowdoin St, 9th Floor, Boston, MA 02114 USA; 3 The Discipline of Psychiatry and Mental Health, School of Clinical Medicine, UNSW Medicine, University of New South Wales Sydney, Sydney, NSW 2052 Australia; 4 Department of Psychology and Neuroscience, University of North Carolina at Chapel Hill, 235 E. Cameron Ave, Room 243, Chapel Hill, NC 27514 USA

**Keywords:** Humanitarian aid, Psychosocial, Workplace stress, Burnout, Mental health, Occupational health, Coping, Path analysis, Bangladesh

## Abstract

**Background:**

While trauma exposure is an established predictor of poor mental health among humanitarian aid workers (HAWs), less is known about the role of psychosocial work-related factors. This study aims to establish a psychosocial model for burnout and psychological distress in HAWs that tests and compares the effects of adversity exposure and workplace stressors in combination, and explores the potential mediating role of individual coping styles.

**Methods:**

Path analysis and model comparison using cross-sectional online survey data were collected from full-time international and local HAWs in Bangladesh between December 2020 and February 2021. HAWs self-reported on exposure to adversities, workplace psychosocial stressors (Third Copenhagen Psychosocial Questionnaire), coping styles (Coping Inventory for Stressful Situations), burnout (Maslach Burnout Inventory—Human Services Survey), and psychological distress (Kessler-6).

**Results:**

Among N = 111 HAWs, 30.6%, 16.4%, 12.7%, and 8.2% screened positive for moderate psychological distress (8 ≤ Kessler-6 ≤ 12), emotional exhaustion (EE ≥ 27), depersonalization (DP ≥ 13), and severe psychological distress (K-6 ≥ 13), respectively. 28.8% reported a history of mental disorder. The preferred model showed distinct pathways from adversity exposure and workplace stressors to burnout, with negative emotion-focused coping and psychological distress as significant intervening variables. While greater exposure to both types of stressors were associated with higher levels of burnout and distress, workplace stressors had a stronger association with psychological outcomes than adversity exposure did (β = .52, *p* ≤ .001 vs. β = .20, *p* = .032). Workplace stressors, but not adversities, directly influenced psychological distress (β = .45, *p* ≤ .001 vs. β = −.01, *p* = .927). Demographic variables, task-focused and avoidance-focused coping were not significantly associated with psychological outcomes.

**Conclusions:**

Compared to exposure to adversities, workplace stressors primarily influenced occupational stress syndromes. Reducing workplace stressors and enhancing adaptive coping may improve psychological outcomes in humanitarian staff.

**Supplementary Information:**

The online version contains supplementary material available at 10.1186/s13031-023-00512-1.

## Background

Given the growing number, scale, and protracted nature of humanitarian emergencies, there is an urgent need to address the mental health of humanitarian aid workers (HAWs) to ensure the quality and sustainability of humanitarian efforts [1, 2]. Most studies on HAW mental health have concentrated on the risks of traumatic stress exposure. Indeed, HAWs operate in high-risk environments of political insecurity, poverty, and natural disasters, often enduring poor living conditions [3]. They also experience or witness potentially traumatic events (PTEs; e.g., attacks, kidnapping, gender-based violence) [4], and may be indirectly exposed to the trauma of the communities they serve [5]. Additionally, HAWs are also often away from family and social support for extended periods of time and may face isolation and acculturation challenges. Exposure to these unique adverse conditions puts HAWs at high risk for concurrent and long-term common mental disorders, such as posttraumatic stress disorder, depression, and anxiety [6–8]. However, the emphasis on exposure to adversities (i.e., poor living conditions, exposure to PTEs, environmental hazards, and deployment-related interpersonal stressors), which are inherent to humanitarian work, neglects the cumulative effects of ongoing daily stressors that can be modifiable.

Complementing the traumatic stress paradigm with an occupational health approach, this study focuses on the psychological effects of chronic psychosocial stressors at the workplace. *Workplace psychosocial stressors* are defined as the “aspects of work design and the organization and management of work, and their social and environmental context, which have the potential to cause psychological or physical harm.” [9, p. 69]. Psychosocial hazards are often categorized into the following ten domains: job content; workload and work pace, work schedule, control over work (e.g., decision making and feedback), environment and equipment, organizational culture and function, interpersonal relationships at work, role and responsibilities in the organization, career development and job security, and home-work interface [10, 11].

In addition, this study focuses on outcomes related to occupational stress syndromes instead of mental health diagnoses. Burnout syndrome, as defined in the International Classification of Diseases (ICD-11) [12], is a consequence of chronic workplace stress that is typically characterized by three dimensions: emotional exhaustion, depersonalization, and reduced personal accomplishment [13]. Emotional exhaustion refers to feelings of being emotionally and physically depleted. Depersonalization touches upon the interpersonal context of burnout and is characterized by excessively distant and cynical attitudes towards service recipients. Reduced personal accomplishment is described by feelings of inefficacy and lack of achievement in one's work. Additionally, non-specific psychological distress is characterized by a constellation of psychological and somatic symptoms common among a wide range of mental disorders [14]. It is a-diagnostic, and cases are distinguished by severity. In a longitudinal study, international staff from international NGOs with higher levels of exposure to chronic stressors were found to have a higher prevalence of emotional exhaustion, depersonalization, depression, and anxiety based on cut-off measures at post-deployment and three-month follow-up, compared to pre-deployment baseline [7]. If unaddressed, burnout and psychological distress among aid workers can lead to a higher accident and illness rates, absenteeism [1, p.7], loss of efficiency and productivity, lower performance, lower work commitment and engagement, and leaving the field of humanitarian work entirely [2, 3].

In addition, the psychological impact from both the potentially traumatic events encountered and occupational environment is likely to be additive. Critically, the interplay between stressors from both the traumatic stress and occupational health paradigms is more likely to explain the observed variations in psychological health outcomes than in isolation [15]. Organizational aspects of work (e.g., workload, financial pressures, lack of recognition for work, difficult relationships with supervisors, and disparity of treatment between international and national staff) were perceived as primary sources of stress across multiple studies with HAWs from United Nations (UN) organizations and other humanitarian contexts [16]. This study will thus be the first to assess and compare the effects of exposure to adversities and workplace psychosocial stressors in combination.

### Understanding the role of coping styles

Given that many risk factors influencing HAW psychological health may be pervasive and difficult to prevent, it is crucial to identify modifiable intervention targets. Coping styles generally refer to a set of coping strategies that remain relatively fixed across time and circumstances, with cumulative evidence suggesting that they can mitigate the relationship between stressful events and one’s mental health [17, 18]. Maladaptive coping styles, such as avoidance-focused coping (engagement in distraction or hedonic activities to maintain momentary well-being) and negative emotional coping (focusing on negative aspects of stressful situations or negative emotions), are associated with greater burnout and psychological distress in various populations [19, 20]. Conversely, task-focused coping (problem-focused orientation where active cognitive and behavioural efforts are used to solve the problem) is positively correlated to a perceived control over stress [21], and has been found to be negatively correlated with anxiety and depression in the general population [22]. Since individuals differ in their response to stressful situations, coping styles may also contribute to variation in psychological outcomes across individuals despite the exposure to the same stressors [23]. However, the limited studies examining coping styles among HAWs have found diverging results [7, 24], which may be explained by a transactional model of stress that posits that that coping efforts are effective based on the goodness-of-fit with the situation at hand [18]. In general, task-focused coping appears to be more effective in controllable situations, whereas emotion-focused coping appears to be more effective in uncontrollable situations [25]. Hence, we can expect that different coping styles may be more or less adaptive depending on the type of stressors faced, providing further support for the need to determine the mediating role of coping style and the varying relationships between stressors, coping styles, and psychological outcomes.

### Humanitarian context of Bangladesh

Bangladesh is a low-income country with high humanitarian need. In August 2017, an estimated 745,000 stateless Muslim Rohingya from the Rakhine state in Myanmar have fled to Cox’s Bazar, making it the largest and fastest refugee influx into Bangladesh [26]. At the same time, Bangladesh’s geographical location also makes it very prone to annual floods and cyclones. In 2021, an astounding count of 1.4 million people in Bangladesh was affected and required humanitarian assistance, served by a total of eight UN agencies, and hundreds of national and international non-governmental organizations (NGOs) [27]. This dire situation had exacerbated during the COVID-19 pandemic. HAWs in Dhaka and Cox’s Bazar face challenging working and living conditions and are classified by the UN as Category C and D duty stations (where E is the most challenging). This will be the first study with HAWs in Bangladesh who provide aid in a post-emergency context with an ongoing refugee crisis amidst the pandemic.

### Study aims

This cross-sectional study aims to establish a psychosocial model that tests the effects of adversity exposure and workplace stressors on burnout and psychological distress in a humanitarian workforce in Bangladesh. We hypothesize that higher levels of exposure to workplace psychosocial stressors and adversity exposure will be associated with higher levels of burnout and psychological distress, respectively. Given results from prior qualitative studies indicating workplace stressors as a primary perceived stressor, we explore the hypothesis that workplace stressors will have a greater effect than adversity exposure on burnout and distress, respectively. The model further generates hypotheses about the potential mediating role of individual coping styles in the relationship between stressors and psychological outcomes.

## Methods

### Study design and participants

An online survey was completed by (N = 111) full-time national and international HAWs in Bangladesh between December 2020 and February 2021. Participants were recruited from various UN agencies, International Federation of Red Cross (IFRC) societies, international and national non-governmental organizations (NGOs) in Bangladesh via convenience and snowball sampling. An invitation email with the survey link was emailed to various sector and inter-sector working group listservs and humanitarian aid organizations human resources/administrative contacts found from organizations' websites. Participants were also encouraged to forward the email and survey link to other colleagues who fit the inclusion criteria, so that more participants may be recruited. Ethical approval was obtained from the Institutional Review Board at Teachers College, Columbia University (Protocol No.: 20-343), and Bangladesh Medical Research Council (Registration No.: 342-28-09-2020).

### Measures

All five measures were self-report questionnaires in the English language. Measures had acceptable to good internal consistency in this sample, as indicated by Cronbach’s alpha, α < 0.7.

*Exposure to adversity* was assessed in a 28-item, original questionnaire, adapted from the Integrated Needs Assessment for United Nations (UN) Staff Survey (Tay, 2020, personal communication) developed from a literature review of common stressors faced by humanitarian aid worker [16]. Four domains were covered: *poor living conditions* (12 items, α = 0.85; e.g., shortage of food/water, lack of adequate health insurance, transport and safety issues, inadequate mobile and internet connection); *deployment-related interpersonal stressors* (five items, α = 0.79; e.g., being separated from family members/friends/community, having difficulties socializing or making friends, acculturation problems); *exposure to potentially traumatic events* (eight items; α = 0.80; e.g., threats of attacks or violence from political instability, bombings/explosions, death or suffering of loved ones/colleagues/persons of concern); and *exposure to environmental hazards* (three items; α = 0.69; e.g., threats of natural or man-made disasters, threats of life-threatening diseases). Participants indicated how frequently they experienced these adversities in the past 12 months on a four-point Likert-scale (1 = “Rarely” to 4 = “Always”). Item scores were summed.

*Workplace psychosocial stressors* were assessed using an adapted 28-item of the Third Copenhagen Psychosocial Questionnaire (COPSOQ-III) [28] commonly used to assess psychosocial risk in workplaces (see Additional file [1]). As there is currently no national version of the COPSOQ-III for Bangladesh, adaptation of the short version of the instrument followed the guidelines set out by the International COPSOQ Network [29]. Items were selected from relevant dimensions of job demands that have been indicated by empirical studies to contribute to HAW psychological well-being [16], including workload, work pace, degree of influence on work tasks, career advancement, role clarity and predictability, recognition at work, organizational justice, fair work distribution, job security, job satisfaction, and work-life conflict. Exposure to sexual harassment, threats of violence, bullying, and discrimination at work were also assessed. In addition, items were standardized to first-person, negatively-worded statements, as advised by local experts for better comprehension of questions and reduce response fatigue. In this study’s sample, the scale had good internal consistency (α = 0.92). Participants rated how frequently they experienced these stressors in the past 12 months on a four-point Likert scale (1 = “Rarely” to 4 = “Always”). Item scores were summed.

*Coping styles* were assessed using the 21-item, short-version of The Coping Inventory for Stressful Situations (CISS-2) [30], which have demonstrated good psychometric properties in many emergency personnel samples across different cultures, including Italian volunteer rescue workers in a natural disaster setting [31]. The instrument consists of three subscales of seven items each: *avoidance-focused coping, negative emotion-focused coping,* and *task-focused coping *[30]*. Avoidance-focused coping* refers to distraction behaviors or social diversion that aim to avoid dealing with the stressful situation directly (α = 0.79; e.g., “I buy myself something.”; “I visit a friend.”). *Emotion-focused coping* indicates that the individual focuses on the negative emotional reactions experienced by individuals when responding to stress (α = 0.88; e.g., “I blame myself for having gotten into this situation.”). *Task-focused coping* entails tackling a stressful situation as a problem to be solved (α = 0.85; e.g., “I focus on the problem and see how I can solve it.”). Participants rated how frequently they use a particular coping strategy on a five-point Likert scale (1 = “Not at all” to 5 = “Very much”). Scores were summed for each coping style.

*Burnout* was assessed using the emotional exhaustion (EE) and depersonalization (DP) subscales from the Maslach Burnout Inventory—Human Services Surveys [13]. The MBI-HSS is the most used tool to assess burnout among professional workers in the field of human services and has been used in many humanitarian aid worker samples [7, 15, 32]. Following previous studies with humanitarian workforces to aid comparison of mean burnout scores, items from only EE and DP subscales were chosen. EE and DP are also more reliable subscales and yield stronger effect sizes compared to the personal accomplishment (PA) subscale in studies with HAW populations [13]. The EE subscale (nine items; α = 0.89) assesses feelings of being emotionally or physically depleted (e.g., “Working with people all day is really a strain for me.”). The DP subscale (five items; α = 0.67) assesses distant and cynical attitudes or behaviors towards service recipients (e.g., “I worry that this job is hardening me emotionally.”). Participants rated how frequently they experienced these feelings in the past 30 days on a seven-point Likert scale (0 = “Never” to 6 = “Everyday”). Following the categorization utilized in the UN Staff Wellbeing Survey [32], a composite score of burnout was generated by summing the scores on both the EE and DP subscales. High risk for burnout is typically associated with scores of 27 or more for EE, and 13 or more for DP [13]. Caseness of burnout in this study was identified by meeting thresholds of both EE and DP.

*Psychological Distress* was assessed using the six-item Kessler-6 (K-6) [33]. The six-item scale is widely used to screen for non-specific psychological distress in the general population and has been validated in many lower- and middle-income countries [30]. In this study’s sample, the scale had good internal consistency (α = 0.85). Participants were asked to rate on a five-point Likert scale how often within the past 30 days they felt: nervous; hopeless; restless or fidgety; depressed; that everything was an effort; and worthless (0 = “Never” to 4 = “All of the time”). Following the polychotomous scoring of the K-6 proposed by Furukawa’s team [34] validated in low-income community populations [35], summed scores of eight to 12 indicate positive screen for moderate psychological distress, and ≥ 13 indicate positive screen for serious psychological distress.

*Demographic information.* Other common predictors of HAW mental health were selected as covariates based on previous literature, including sex, age, nationality, employment level, organization type, length of employment, length of humanitarian aid service, previous psychiatric history, whether they worked with people of concern, and whether they lived at the duty station.

### Data analysis

To compare means of variables across socio-demographic groups, *t*-tests, Brown-Forsythe tests for unequal variances, and one-way analysis of variance (ANOVA) were performed using SPSS (Version 27). Established thresholds were used to interpret effect sizes, where Cohen’s *d* of 0.2, 0.5, and 0.8 denoted small, medium, and large effects, respectively [36]. Significant demographic variables will be controlled for in subsequent path models.

**Fig. 1:**
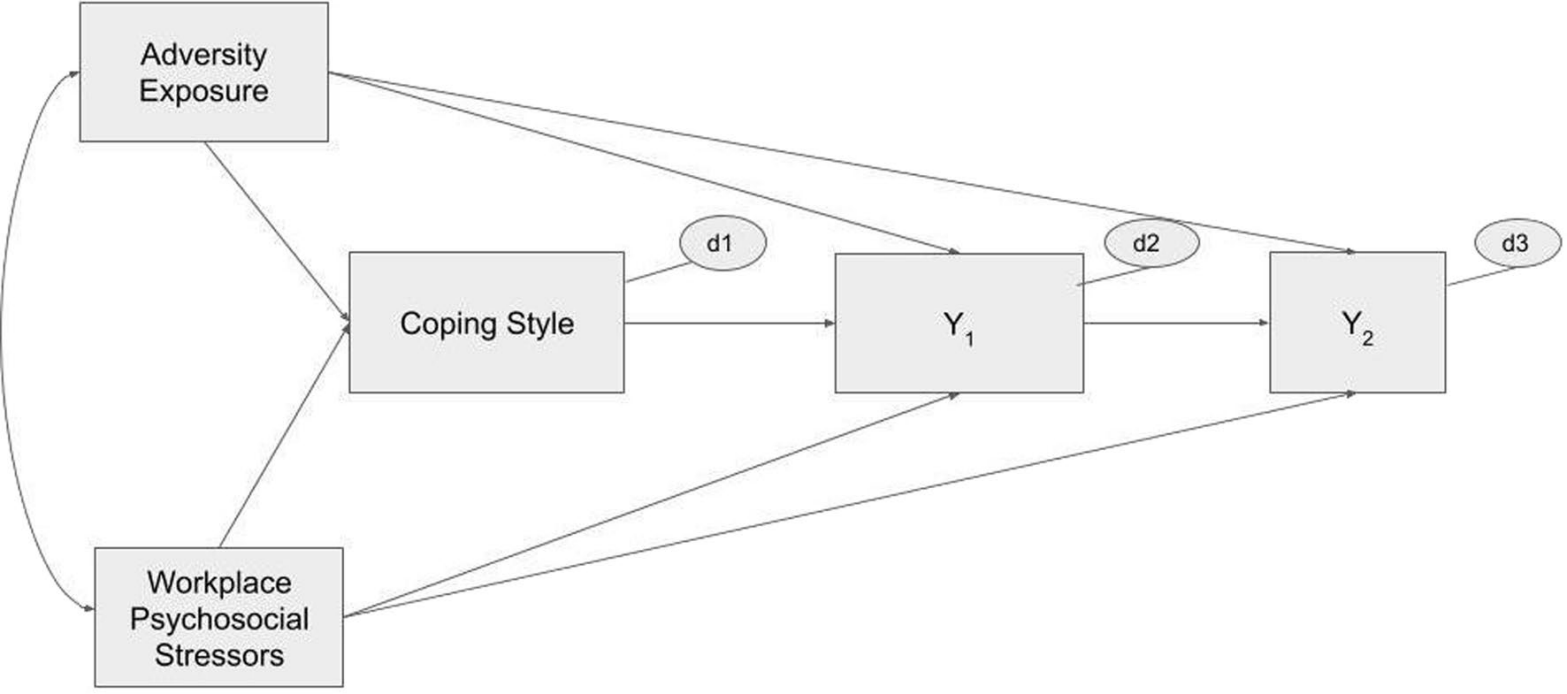
Hypothesized Path Models for Burnout and Psychological Distress. *Note.* One-way arrows represent paths; two-way arrows represent covariances; *d* represents disturbances of endogenous variables (i.e., all unmeasured exogenous variables, random, and measurement errors)

A stepwise process of model building using path analysis was used to determine a theory-driven model with risk pathways to burnout and psychological distress. Two path models were analyzed: with burnout as outcome (Y_2_) and distress as intervening variable (Y_1_), and vice versa (see Fig. 1).

Both models tested direct paths from adversity exposure and workplace stressors to psychological outcomes, as well as their indirect effects through respective coping styles. Models were tested with observed continuous variables and maximum-likelihood estimation, bootstrapped for 5,000 replications, using SPSS AMOS (Version 26). Missing values were handled with regression imputation. Significant effects were supported when zero falls outside of the 95% bias-corrected bootstrap confidence intervals (95% CI). Non-significant paths were trimmed. Final models were compared on several exact and approximate fit indices [30], including: a non-significant chi-square (*χ*^*2*^); Comparative Fit Index (CFI) value > 0.90; Root Mean Square Error of Approximation (RMSEA) value < 0.08; and maximum likelihood-based Standardized Root Mean Residual (SRMR) value of ≤ 0.08.

## Results

### Participants

Sociodemographic characteristics of the sample are summarized in Table 1.

**Table 1:** Sociodemographic characteristics of participants

Sociodemographic characteristic	Full sample (*N* = 111)	
	*n*	%
Sex		
Male	64	57.7
Female	47	42.3
Nationality		
Bangladeshi	99	89.2
International	12	10.8
Job level		
Full-time aid worker	26	23.4
Middle-manager	55	49.5
Executive leadership	30	27.0
Organization type		
UN Agencies/IFRC Societies	22	19.8
International NGO	42	37.8
National NGO	47	42.3
Direct work with persons of concern^a^	80	72.1
Lives at duty station^a^	51	45.9
Previous history of mental disorder^a,b^	32	28.8

^a^Reflects participants answering “Yes” to this question

^b^*n* = 7 preferred not to disclose their psychiatric history and were coded as missing values

HAWs from 52 aid organizations responded to the survey with less than 15% incomplete data (N = 111). The sample included 64 men (57.7%) and 47 women (42.3%), with a mean age of 37.4 years old (SD = 10.7). Most of them were Bangladeshi HAWs (n = 99, 89.2%). Approximately two-fifths of the sample came from national NGOs (n = 47, 42.3%) and international NGOs (n = 55, 37.7%), while one-fifth came from UN or IFRC agencies (19.8%, n = 22). Participants had spent an average of 47 months (SD = 69.1) working in their current organization and 75 months (SD = 98.3) in the humanitarian sector. About half of the sample were middle-managers (n = 55, 49.5%), while approximately one-quarter were full-time staff (n = 26, 23.4%) or executive leaders (n = 30, 27.0%). Participants came from a variety of aid sectors, including: Emergency and Disaster Management and Response; Health; Monitoring, Evaluation, Accountability and Learning; Protection (Child Protection and Gender-Based Violence); and Water, Sanitation, and Hygiene sectors. Most of the participants worked directly with persons of concern (n = 80, 72.1%), and about half lived at the duty station (n = 51, 45.9%). Close to a third of the participants (n = 32, 28.8%) reported having a previous history of mental disorder.

### Burnout and psychological distress

Based on the cut-offs in the literature, 30.6% (n = 34), 16.4% (n = 18), 12.7% (n = 14), and 8.2% (n = 9) screened positive for moderate psychological distress, emotional exhaustion, depersonalization, and severe psychological distress, respectively. 8.2% (n = 9) met the cut-offs for both emotional exhaustion and depersonalization. HAWs who reported a history of mental disorder reported higher scores of psychological distress (M = 9.1, SD = 5.2, N = 32) than those who did not have a history of mental disorder (M = 5.7, SD = 3.9, N = 71) with medium effect size, *t* (101) = -3.68, *p* < 0.001, *d* = 0.78. Other demographic variables did not have a significant effect on burnout and psychological distress.

### Exposure to adversity

The median number of potentially stressful life events experienced was 14 out of 28 (SD = 6.5). Specifically, the top five most frequently cited perceived adversities were: worrying about the well-being of family members (86.6%); being separated from family members due to work responsibilities (81.4%); threats of life-threatening or deadly diseases (e.g., COVID-19) (80.4%); travel difficulties (78.4%); and feeling isolated (74.2%) (Additional file 2). Staff with a history of mental disorder also endorsed higher frequency of exposure to common adversities overall (M = 52.5, SD = 11.2, n = 25) compared to staff without a mental disorder history (M = 45.9, SD = 10.2, n = 66), with medium effect size, *t*(89) = -2.67, *p* = 0.009, *d* = 0.63.

### Workplace psychosocial stressors

The mean number of workplace stressors experienced was 17 out of 28 (SD = 6.5). In terms of *offensive behaviors at work*, 6.1%, 13.3%, and 34.7% reported experiencing some form of sexual harassment, violence, and bullying at work, respectively. Close to half of the sample (n = 47, 48.5%) reported experiencing discrimination at work, including discrimination due to gender (n = 19, 19.6%), age (n = 10, 10.3%), race/ethnicity (n = 6, 6.2%), nationality (n = 6, 6.2%), sexual orientation (n = 4, 4.1%), pregnancy/parenthood (n = 4, 4.1%), and mental health (n = 1, 1.0%). Exposure to workplace stressors did not differ across demographic groups.

### Coping styles

Participants endorsed more use of task-focused coping strategies (M = 24.93, SD = 5.30), followed by avoidance-focused (M = 19.73, SD = 5.17), and negative emotion-focused coping strategies (M = 18.92, SD = 6.26). Staff with psychiatric illness history reported greater use of negative emotion-focused coping strategies (M = 21.8, SD = 7.1, N = 24) than those who did not have psychiatric history (M = 17.8, SD = 5.7, N = 68), with medium effect size, *t*(88) = −2.76, *p* = 0.007, *d* = 0.66. Task- and avoidance-focused styles were not significantly associated with any demographic variables.

### Psychosocial model

Correlations between variables are reported in Table 2. As avoidance-focused coping and task-focused coping were not significantly associated with either psychological health outcomes in bivariate analyses, only negative emotion-focused coping was tested as an intervening variable in the subsequent path models. As bivariate analyses found that past psychiatric history was significantly associated with study variables, we controlled for history of mental illness in subsequent path models.

**Table 2:** Descriptive statistics and Pearson’s correlations for main variables

Scale	MBI-HSS	K-6	Adversity Exposure	COPSOQ-III	Avoidance-focused Coping		Emotion-focused Coping	Task-focused coping
MBI-HSS	–							
K-6	.63**	–						
Adversity Exposure	.42**	.39**	–					
COPSOQ-III	.57**	.65**	.50**	–				
Avoidance-focused coping	.15	.13	.17	.10	–			
Emotion-focused coping	.46**	.60**	.48**	.54**	.22*	–		
Task-focused coping	.06	.03	.13	.08	.40**	.25*		–
*N*	110	110	97	97	96	96		96
Mean	21.0	6.8	48.0	52.4	19.7	18.9		24.9
*SD*	15.6	4.6	11.1	12.8	5.2	6.3		5.3
Actual range	0–78	0–24	27–81	30–93	7–31	7–35		7–34
α	.90	.85	.90	.92	.79	.88		.85

*MBI-HSS*: Maslach Burnout Inventory—Human Services Scale, *K-6*: Kessler-6 scale, *COPSOQ-III*: Third Copenhagen Psychosocial Questionnaire (Adapted)

*p < .05, two-tailed; **p < .001, two-tailed

**Fig. 2:**
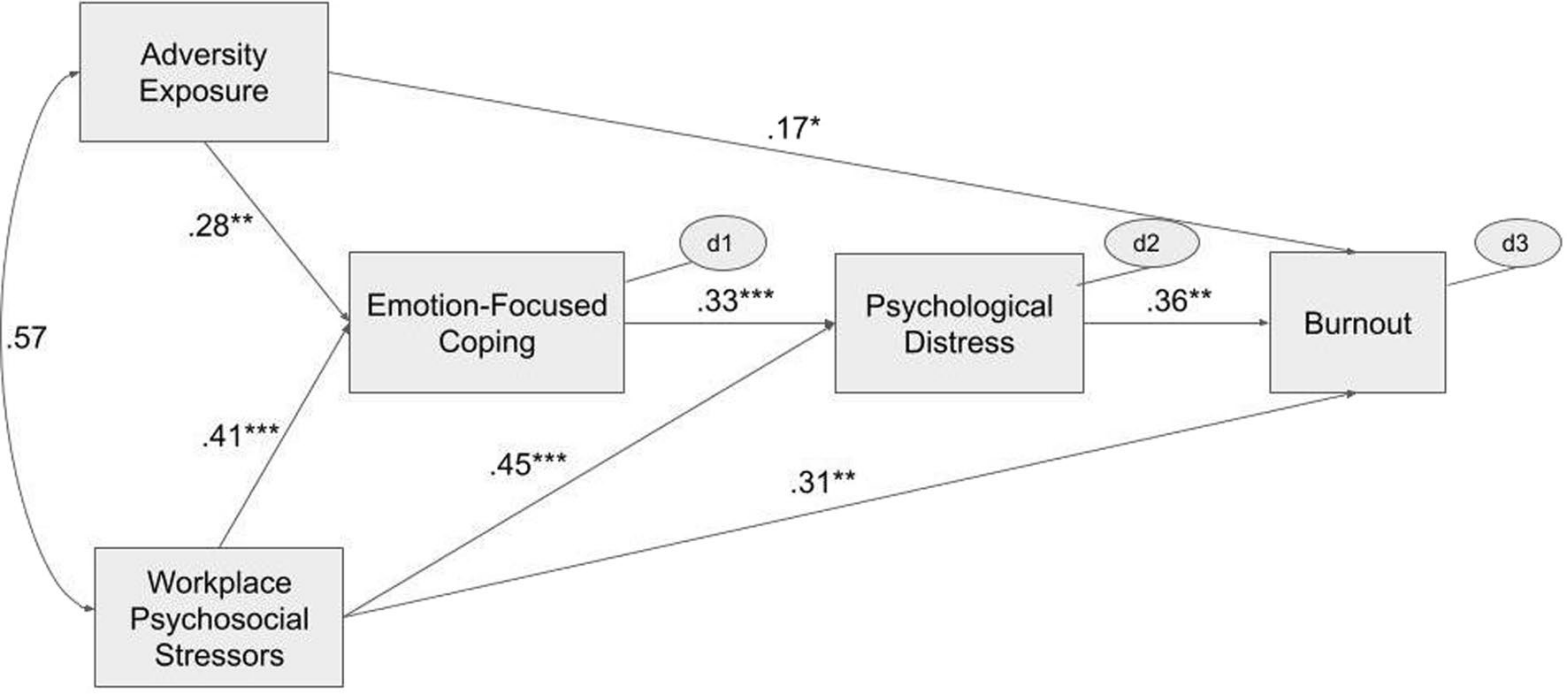
Psychosocial Model of Burnout among Humanitarian Aid Workers in Bangladesh. *Note. N* = 111, *n* = 5000 bootstrap replications. Coefficients standardized. Non-significant paths removed. One-way arrows represent paths; two-way arrows represent covariances; d represents disturbances of endogenous variables (i.e., all unmeasured exogenous variables, random, and measurement errors). *p < .05; **p < .01; ***p < .001

After comparing model fit statistics (Table 3), the psychosocial model for burnout was preferred (Fig. 2). The model with burnout as the outcome (i.e., distress as an intervening variable) had good exact fit, *X*^2^(2) = 0.733, *p* < 0.693, while the model with distress as the outcome had poor exact fit indicated by significant chi-square value, *X*^2^(3) = 13.423, *p* < 0.004. The model for burnout also had better approximate fit indices than the model for distress, as indicated by higher CFI (1.027 vs. 0.957), lower RMSEA (0.000 vs. 0.178), and lower SRMR (0.011 vs. 0.049). The decomposition of effects of the integrated model for burnout is summarized in Table 4 (see ‘Additional file 3’ for rejected model).

**Table 3:** Fit statistics for psychosocial models for burnout and distress

Fit statistic	Outcome	
	Burnout	Psychological distress
χ^2^_M_	0.733	13.423
*df*_M_	2	3
*p*	.693	.004
CFI	1.027	.957
RMSEA [90% CI]	.000 [.000, .140]	.178 [.089, .279]
SRMR	.011	.049

Fit statistics are reported for final models with non-significant paths removed

*χ*^*2*^_*M*_: likelihood ratio chi-square, df_M_: model degrees of freedom, *CFI*: Comparative Fit Index, *RMSEA:* root mean square error of approximation, *CI*: confidence interval, *SRMR*: standardized root mean residual

**Table 4:** Decomposition of effects from psychosocial model for burnout

Effect	β	*B*	SE	95% CI [LL, UL]	*p*
Direct effects					
Adversity Exposure → EmoCope	.28	0.18	0.06	[0.05, 0.29]	.002
Workplace Stressors → EmoCope	.41	0.20	0.05	[0.12, 0.30]	< .001
Adversity Exposure → Distress	−.01	−0.01	0.05	[−0.09, 0.09]	.927
Workplace Stressors → Distress	.45	0.16	0.04	[0.09, 0.24]	< .001
EmoCope → Distress	.33	0.24	0.06	[0.13, 0.37]	< .001
Adversity Exposure → Burnout	.17	0.27	0.13	[0.01, 0.53]	.039
Workplace Stressors → Burnout	.31	0.38	0.15	[0.10, 0.69]	.007
Distress → Burnout	.36	1.23	0.47	[0.30, 2.14]	.010
Indirect effects					
Adversity Exposure → Burnout via EmoCope and Distress	.05	0.05	0.03	[0.01, 0.14]	.006
Adversity Exposure → Burnout via Distress	−.01	−0.01	0.06	[−0.13, 0.12]	.871
Workplace Stressors → Burnout via EmoCope and Distress	.06	0.05	0.04	[0.01, 0.17]	.005
Workplace Stressors → Burnout via Distress	.20	0.19	0.10	[0.05, 0.43]	.006
EmoCope → Burnout via Distress	.12	0.30	0.15	[0.06, 0.66]	.007
Adversity Exposure → Distress via EmoCope	.09	0.04	0.02	[0.01, 0.09]	.004
Workplace Stressors → Distress via EmoCope	.14	0.05	0.02	[0.02, 0.09]	< .001
Total effects					
Adversity Exposure → Burnout	.20	0.31	0.15	[0.03, 0.59]	.032
Workplace Stressors → Burnout	.52	0.64	0.12	[0.42, 0.90]	< .001
EmoCope → Burnout	.12	0.30	0.15	[0.06, 0.66]	.007
Distress → Burnout	.34	1.23	0.47	[0.30, 2.14]	.010

Outcome variable	*R*^*2*^	*SE*	95% CI [LL, UL]	*p*
Negative emotion-focused coping	.38	.07	[0.22, 0.51]	.001
Psychological distress	.46	.10	[0.26, 0.63]	.001
Burnout	.50	.08	[0.31, 0.64]	.002

*N* = 111, *n* = 5000 bootstrap replications.

*SE*: bootstrap standard error, *EmoCope*: negative emotion-focused coping, *CI*: bias-corrected bootstrap confidence interval, *LL*: lower limit, *UL*: upper limit

Adversity exposure and workplace stressors had overlapping but distinct pathways to burnout. Both types of stressors affected burnout directly, and indirectly through negative emotion-focused coping, while controlling for psychiatric history. The total indirect effect of both stressors on burnout through negative emotion-focused coping was β = 0.12, 95% CI [0.06, 0.66], *p* = 0.007. However, workplace stressors but not adversity exposure had a direct effect on psychological distress (β = 0.45, 95% CI [0.09, 0.24], *p* ≤ 0.001 vs. β = −0.01, 95% CI [−0.09, 0.09], *p* = 0.927).

In addition to having more direct and indirect pathways to burnout, the effects of workplace stressors also had the greatest magnitude. The total effect from workplace stressors to burnout was β = 0.52, SE = 0.12, 95% CI [0.42, 0.90], *p* ≤ 0.001, which was of greater than the total effects from psychological distress to burnout, β = 0.34, SE = 0.47, 95% CI [0.30, 2.14], *p* = 0.010, and adversity exposure to burnout, β = 0.20, SE = 0.15, 95% CI [0.03, 0.59], *p* = 0.032.

The model explained 38% of the variance in emotion-focused coping (SE = 0.07, 95% CI [0.22, 0.51], *p* = 0.001); 46% of the variance in psychological distress (SE = 0.10, 95% CI [0.26, 0.63], *p* = 0.001); and 50% of the variance in burnout (SE = 0.08, 95% CI [0.31, 0.64], *p* = 0.002).

## Discussion

This is the first study to establish an empirically-supported psychosocial model of burnout that examined the effects of adversity exposure and workplace stressors unique to HAWs in a previously unsampled post-emergency context of Bangladesh. Results demonstrated that workplace stressors were greater drivers of burnout compared to adversity exposure. Negative emotion-focused coping was a significant intervening variable in the relationship between stressors and psychological health.

Similar to the rates found in other studies with HAWs [16], the rate of burnout and psychological distress among HAWs in Bangladesh was substantial, with 30.9% of the sample screening positive for moderate psychological distress and 8.2% screening positive for burnout and serious psychological distress, respectively. Notably, only past psychiatric history was a statistically significant predictor of burnout and psychological distress in this sample, which was controlled for in the path models. Contrary to qualitative findings from other studies that highlighted the greater risk of exposure to workplace stressors and adversities among field workers, national, and female staff [3, 16], these sociodemographic variables were not statistically significant predictors of exposure to stressors or psychological outcomes in this study. These findings together is consistent with results from a recent systematic review that history of mental disorder is one of the few reliable predictors of psychological distress and disorder in this occupation group [37]. While unique stressors faced by different demographic groups should be paid attention to, these convergent statistical results may also reflect a generally high level of resilience among these groups.

The preferred model indicated that non-specific psychological distress might precede burnout, which supports the theory that more context-general psychological distress may translate into a negative assessment of one’s work situation and may decrease the individual’s access to psychological resources to meet job demands [38, 39].

The model further indicated that workplace psychosocial stressors had a greater influence on psychological outcomes than adversity exposure, underscoring the importance of addressing workplace stressors as primary sources of occupational stress syndromes in humanitarian staff [2, 40, 41]. One possible explanation is that work organization and communication stressors such as lack of role clarity and lack of recognition at work can contribute to low job control [42], which in turn strongly predicts mental health outcomes and burnout among humanitarian staff [15]. Low personal rewards and sacrifices of demanding humanitarian work may also contribute to a high effort-reward imbalance [43], and thus poor mental health outcomes and burnout [1, 15].

Supporting this study’s finding that adversity exposure did not directly influence psychological distress, a recent study with humanitarian and development workers also found that the number of potentially traumatic events experienced by aid workers was not associated with poor mental health outcomes [44]. Aid workers may be more able to accept or more desensitized to exposure to potentially traumatic or stressful life events that they perceive as inherent to aid work [45]. Another potential explanation could be that the meaningfulness of HAWs’ work has a buffering protective role against psychological stressors arising from humanitarian work [44]. Additionally, Bangladesh’s post-emergency context may also have relatively less exposure to common adversities than high-conflict humanitarian contexts.

Finally, the psychosocial model also supported the hypothesis that greater use of negative emotion-focused coping strategies was the indirect pathway through which workplace stressors and common adversities affected burnout and distress, adding to the established evidence base [46, 47]. Notably, avoidance-focused coping and task-focused coping strategies were not significantly related to psychological outcomes, consistent with findings from national humanitarian staff from Sri Lanka and South Sudan [7, 48]. This result may be an artifact of the measure, where the emotion-focused coping subscale consistently has the strongest association with poor psychological outcomes compared to the other subscales [30]. Moreover, since coping strategies are context-dependent [18], problem-solving and disengagement strategies may be less appropriate and accessible coping methods than emotion-focused strategies in an emergency context where individuals may be powerless to change or avoid stressors actively.

### Limitations

The study presents limitations related to the timing of assessment, its cross-sectional design, sampling, and measurement. First, since data were collected during the start of the COVID-19 pandemic, our findings may have indicated inflated exposure to work stress and levels of burnout and psychological distress [49]. The increased health risk of an infectious disease compounded with the adverse consequences of lockdowns may have exacerbated already resource-poor living conditions, interpersonal stressors (e.g., maintaining social support, ability to visit family), and workplace stressors (e.g., increased workload, diminished work-life balance from working from home). As more data are published about rates of burnout and distress among other frontline workers during the COVID-19 period, future studies will be enriched with a comparative sample.

Secondly, as this was a cross-sectional study, we are limited in our ability to draw causal inferences about the relationships between the risk factors and psychological outcomes. While both theory and empirical evidence provide strong support for the psychosocial model tested, future research should take a longitudinal approach to determine directionality and make current findings more robust. Thirdly, the self-selected, English-speaking, majority Bangladeshi sample in the current study may limit the generalizability of our findings to a large and diverse global population of aid workers. The constrained sample may have precluded finer-grained analyses of the key differences across socio-occupational groups. In addition, the rates of burnout and distress in this study may be elevated since close to 90% of the sample were Bangladeshi humanitarian aid workers and national staff face more security risks due to increasing localization of aid [4]. While we focused only on full-time HAWs in this study, future studies would be wise to also compare outcomes in part-time HAWs, a substantial proportion of the humanitarian workforce, since these two groups may encounter unique challenges due to various resource distributions or different job requirements.

There are also measurement limitations pertaining to self-report, the instruments, and the scoring approach. There may be underreporting in self-report due to difficulty in recognizing burnout, self-stigma, and minimizing of symptoms. Secondly, future studies can improve the cultural validity of instruments with qualitative research on cross-cultural understandings of burnout and organizational culture, as well as with translation and psychometric validation of scales. Thirdly, due to the insufficient sample size, the measurement component of structural equation modeling could not be carried out to test common adversities, workplace stressors, and psychological outcomes as latent variables. Future studies should also consider alternative explanations of factors, as supported by theory and measurement models. Specifically for the measurement of burnout, a key limitation was the exclusion of the Personal Accomplishment dimension of burnout, which may be a possible moderator of burnout and distress [50]. Future studies can consider including possible protective factors, such as having a sense of meaning and social support, to build a more comprehensive model. Furthermore, while this study decided to use established cut-offs for the burnout subscales to facilitate comparison with rates found in other studies with humanitarian workforces, future studies may utilize a more nuanced approach to investigate and delineate distinct burnout profiles present among humanitarian aid workers [51].

### Implications

The study’s findings can inform recommendations for mental health and psychosocial support of HAWs. While existing studies have often proposed pre-deployment screening to prevent psychological disorders in HAWs during and post-deployment, this study makes recommendations for intervening with modifiable factors. Firstly, humanitarian organizations can strive to reduce workplace psychosocial stressors that are salient for humanitarian staff (e.g., organizational culture and interpersonal relationships at work, work organization and communication, and work-life stressors). For instance, manager training can foster team-building and appreciation and enhance fair distribution and communication of work responsibilities [52]. Increasing available benefits, such as more time-off, confidential and accessible health and mental health care, can significantly benefit job satisfaction and improve work-life balance [24]. Secondly, since emotion-focused coping was a significant intervening variable between stressors and psychological outcomes, peri-deployment psychoeducational and stress management training in organizations can promote adaptive coping strategies [53]. Further qualitative and mixed methods research can explore the specific workplace stressors salient to different demographic groups of HAWs, as well as the types of negative emotions elicited by different types of stressors to guide targeted intervention efforts.

## Conclusions

This pioneering study established a comprehensive psychosocial framework on the risk pathways to burnout in a humanitarian workforce in Bangladesh. While about two-thirds of aid workers in this sample were in the healthy range of psychological functioning, the rates of occupational distress were still substantial and worth addressing. The results underscored the primary influence of modifiable workplace psychosocial stressors over adversity exposure on occupational stress syndromes and further suggested the potential mediating role of negative emotion-focused coping between stressors and psychological health. Targeted interventions that reduce workplace psychosocial stressors and enhance adaptive coping are likely to prevent and alleviate burnout in HAWs. Future mixed methods studies investigating effects of specific types of adversities, workplace stressors, and types of negative emotions on psychological outcomes can guide more nuanced intervention efforts. Further replication studies and comparative analyses with more representative and larger samples of staff in various humanitarian settings, organizations, and cultures are encouraged to build a comprehensive understanding of HAW occupational mental health.

## Supplementary Information

**Additional file 1**. Third Copenhagen Psychosocial Questionnaire - Adapted.

**Additional file 2**. Percentage reporting Frequency of Exposure to Types of Adversities.

**Additional file 3.** Psychosocial Model and Decomposition of Effects for Psychological Distress.

## Data Availability

The data that support the findings of this study are available from the corresponding author, CF, upon reasonable request.

## Abbreviations

DP: Depersonalization (burnout dimension)
EE: Emotional exhaustion (burnout dimension)
K-6: Kessler-6
HAW: Humanitarian aid worker
NGO: Non-governmental organizations
UN: United Nations
