# Supplementary material for: Psychosocial model of burnout among humanitarian aid workers in Bangladesh: role of workplace stressors and emotion coping
Source: Confl Health. 2023 Apr 3;17:17. doi: 10.1186/s13031-023-00512-1 (PMC10068704; doi:10.1186/s13031-023-00512-1)
Supplement: Supplementary file 1 — Additional file 1. Third Copenhagen Psychosocial Questionnaire - Adapted. [file 13031_2023_512_MOESM1_ESM.pdf]

Additional file 1

COPSOQ III - Adapted

The following questions are about your psychosocial work environment. Please answer with the option that best fits your view.

		**Never**	**Sometimes**	**Often**	**Always**
**1.**	**I do not have time to complete all my work tasks.**	0	1	2	3
**2.**	**I struggle with meeting deadlines.**	0	1	2	3
**3.**	**I have difficulties keeping up with the pace of work.**	0	1	2	3
**4.**	**I have to work at a high pace throughout the day.**	0	1	2	3
**5.**	**I have to deal with other people's personal problems as part of my work.**	0	1	2	3
**6.**	**I find my work emotionally demanding.**	0	1	2	3
**7.**	**I find that my work requires me to hide my feelings.**	0	1	2	3
**8.**	**I do not have a large degree of influence on the decisions concerning my work.**	0	1	2	3
**9.**	**I cannot influence the amount of work assigned to me.**	0	1	2	3
**10.**	**I do not learn new skills and knowledge through my work.**	0	1	2	3
**11.**	**I am asked to perform duties outside of my professional training.**	0	1	2	3
**12.**	**I spend too much time on administrative tasks and not enough time on my core duties.**	0	1	2	3
**13.**	**I have difficulties advancing**	0	1	2	3

	**my career.**				
**14.**	**I have to do overtime.**	0	1	2	3
**15.**	**I do not find my work meaningful.**	0	1	2	3
**16.**	**I feel that the work I do is not important.**	0	1	2	3
**17.**	**At my workplace, I am informed at the last minute about important decisions, changes or plans for the future.**	0	1	2	3
**18.**	**I receive very little direction and information from the management/supervisor to do my work well.**	0	1	2	3
**19.**	**I am not sure what is expected of me at work.**	0	1	2	3
**20.**	**My work is not recognized nor appreciated by the management/supervisor.**	0	1	2	3
**21.**	**I am worried about becoming unemployed.**	0	1	2	3
**22.**	**I am worried about being transferred to another job or mission against my will.**	0	1	2	3
**23.**	**I feel that my work drains so much of my <u>energy</u> that it has a negative effect on my private life.**	0	1	2	3
**24.**	**I feel that my work takes so much of my <u>time</u> that it has a negative effect on my private life.**	0	1	2	3
**25.**	**Conflicts and misunderstandings in the workplace are not resolved in a fair way.**	0	1	2	3
**26.**	**Work is not distributed fairly.**	0	1	2	3

**27.**	**I am pleased with my job as a whole after taking everything into consideration.**	0	1	2	3
**28.**	**I receive untoward sexual advances at my workplace.**	0	1	2	3
	If response is 1, 2, or 3 for Q27, follow up with: From whom did you receive untoward sexual advances from? (multiple response options): Colleagues; Manager/supervisor; Subordinates; Beneficiaries/clients; Do not want to say				
**29.**	**I am exposed to threats of violence at my workplace.**	0	1	2	3
	If response is 1, 2, or 3 for Q28, follow up with: What kind of violence were you exposed to? (multiple response options): Physical violence; Verbal abuse; By colleagues; By manager/supervisor; By subordinates; By beneficiaries/clients; Do not want to say				
**30.**	**I am exposed to bullying (i.e., unpleasant and degrading treatment) at my workplace.**	0	1	2	3
	If response is 1, 2, or 3 for Q29, follow up with: Who were you bullied by? (multiple response options): Colleagues, Manager/supervisor, Subordinates, Beneficiaries/clients, Do not want to say.				
**31.**	**I have a difficult time at work because of discrimination.**	0	1	2	3
	If response is 1, 2, or 3 for Q30, follow up with: I faced discrimination because of my (multiple response options): Physical disability; Mental health problems; Race and/or ethnic group; Nationality; Age; Gender; Sexual orientation; Pregnancy or parenthood; Other (please specify); Do not want to say				
