# Supplementary material for: Psychosocial model of burnout among humanitarian aid workers in Bangladesh: role of workplace stressors and emotion coping
Source: Confl Health. 2023 Apr 3;17:17. doi: 10.1186/s13031-023-00512-1 (PMC10068704; doi:10.1186/s13031-023-00512-1)
Supplement: Supplementary file 2 — Additional file 2. Percentage reporting Frequency of Exposure to Types of Adversities. [file 13031_2023_512_MOESM2_ESM.pdf]

Additional file 2

Percentage reporting Frequency of Exposure to Types of Adversities

Type of Adversity	%
Poor Living Conditions	
Travel difficulties due to safety restrictions and road conditions	78.4
Lack of access to recreational activities	68.1
Lack of access to mental health care	66.1
Inadequate mobile and internet connection	62.9
Lack of adequate health insurance	57.7
Financial concerns	53.6
Lack of access to primary care and specialist treatments	53.6
Lack of privacy (e.g., shared sanitary, shower facilities)	36.1
Lack of access to basic amenities (e.g., shelter, bank, mosque, church)	34.0
Shortage of food and/or clean water	32.0
Lack of personal protective equipment (e.g., surgical masks, scrubs)	29.9
Interpersonal Stressors	
Worrying about wellbeing of family members/children	86.6
Having difficulties socializing or making new friends	72.2
Feeling isolated	74.2
Being separated from family members, friends, community due to work	81.4
Acculturation or adjustment problems	66.0
Exposure to PTEs	
Missing or lost loved ones/friends/colleagues	58.8
Death of loved ones/friends/colleagues	51.5
Witnessing suffering or death of persons of concern	49.5
Gender-based violence	42.3
Having to flee suddenly or displacement	25.8
Threats of malicious acts (e.g., armed robbery, carjacking)	21.7
Threats of attacks or violence due to political instability (e.g., armed militia)	11.4
Shellings, bombings, or explosions	8.3
Environmental Stressors	
Threats of life-threatening or deadly diseases (e.g., COVID-19)	80.4
Extreme weather conditions (e.g., heat, rain, cold)	73.2
Threats of environmental, natural, or man-made hazards (e.g., landslide)	59.8

Note. Percentages reflect those who reported moderate and high frequency of exposure to types of common adversities, in descending order. $N = 97$.
