# Supplementary material for: Psychosocial model of burnout among humanitarian aid workers in Bangladesh: role of workplace stressors and emotion coping
Source: Confl Health. 2023 Apr 3;17:17. doi: 10.1186/s13031-023-00512-1 (PMC10068704; doi:10.1186/s13031-023-00512-1)
Supplement: Supplementary file 3 — Additional file 3. Psychosocial Model and Decomposition of Effects for Psychological Distress. [file 13031_2023_512_MOESM3_ESM.pdf]

Additional file 3

Psychosocial Model for Psychological Distress

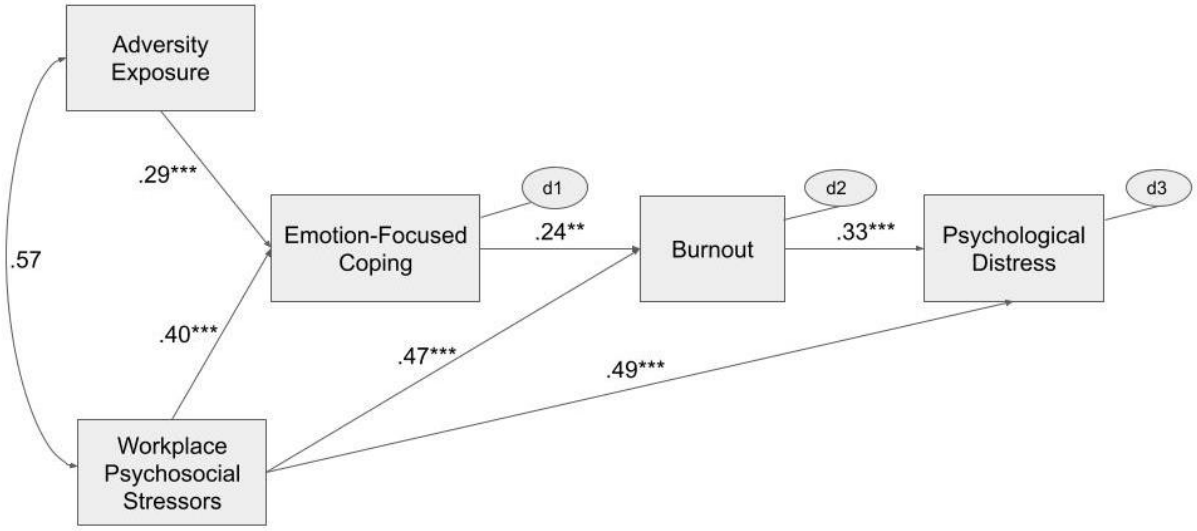

Note. Model fit was poor and model was not preferred over integrated model for burnout. $N = 111$, $n = 5{,}000$ bootstrap replications. Coefficients standardized. Non-significant paths removed. One-way arrows represent paths; two-way arrows represent covariances; *d* represent disturbances of endogenous variables (i.e., all unmeasured exogenous variables, random, and measurement errors).

* $p < .05$; ** $p < .01$; *** $p < .001$.

Decomposition of Effects from Psychosocial Model for Psychological Distress

Effect	β	*B*	SE	95% CI [LL, UL]	*p*
Direct Effects					
Adversity Exposure → EmoCope	.29	0.18	0.06	[0.06, 0.29]	.006
Workplace Stressors → EmoCope	.40	0.19	0.05	[0.11, 0.29]	< .001
Adversity Exposure → Burnout	.41	0.25	0.15	[-0.05, 0.56]	.114
Workplace Stressors → Burnout	.15	0.50	0.13	[0.26, 0.77]	< .001
EmoCope → Burnout	.19	0.49	0.24	[0.04, 0.96]	.035
Adversity Exposure → Distress	.01	< 0.01	0.04	[-0.08, 0.09]	.936
Workplace Stressors → Distress	.49	0.17	0.05	[0.09, 0.27]	.001
Burnout → Distress	.33	0.10	0.03	[0.03, 0.16]	.007
Indirect Effects					
Adversity Exposure → Distress via EmoCope and Burnout	.02	0.01	0.01	[0.01, 0.03]	.011
Adversity Exposure → Distress via Burnout	<.01	0.02	0.02	[0.00, 0.07]	.048
Workplace Stressors → Distress via EmoCope and Burnout	.03	0.01	0.01	[0.01, 0.03]	.020
Workplace Stressors → Distress via Burnout	.05	0.05	0.02	[0.01, 0.10]	.004
EmoCope → Distress via Burnout	.06	0.05	0.03	[0.01, 0.12]	.026
Adversity Exposure → Burnout via EmoCope	.06	0.09	0.05	[0.02, 0.21]	.015
Workplace Stressors → Burnout via EmoCope	.08	0.09	0.06	[0.01, 0.23]	.027
Total Effects					
Adversity Exposure → Distress	.07	0.04	0.05	[-.06, 0.13]	.427
Workplace Stressors → Distress	.65	0.23	0.05	[0.14, 0.32]	.001
EmoCope → Distress	.06	0.05	0.03	[0.01, 0.12]	.026
Burnout → Distress	.33	0.10	0.03	[0.03, 0.16]	.007

Outcome Variable	R^2	*SE*	95% CI [LL, UL]	*p*
Negative Emotion-Focused Coping	.38	0.08	[0.22, 0.52]	.001
Burnout	.42	0.10	[0.19, 0.58]	.001
Psychological Distress	.55	0.09	[0.33, 0.69]	.001

Note. *N* = 111, bootstrapped for 5,000 replications. *SE*: bootstrap standard error. EmoCope: negative emotion-focused coping. CI: bias-corrected bootstrap confidence interval. LL: lower limit. UL: upper limit.
